# A Systematic Review and Meta-Analysis of Front-line Anthracycline-Based Chemotherapy Regimens for Peripheral T-Cell Lymphoma

**DOI:** 10.5402/2011/623924

**Published:** 2011-06-16

**Authors:** Abeer N. AbouYabis, Pareen J. Shenoy, Rajni Sinha, Christopher R. Flowers, Mary Jo Lechowicz

**Affiliations:** ^1^Department of Internal Medicine, Mercer University, Central Georgia Cancer Care, 1062 Forsyth Street, Suite 1B Macon, Georgia, GA 31201, USA; ^2^Department of Hematology Oncology, Winship Cancer Institute, Emory University, 2365 Clifton Road, N.E. Building C, Atlanta, GA 30322, USA

## Abstract

Anthracycline-based chemotherapy remains standard treatment for peripheral T-cell lymphoma (PTCL) although its benefits have been questioned. We performed systematic literature review and meta-analyses examining the complete response (CR) and overall survival (OS) rates for patients with PTCL. The CR rate for PTCL patients ranged from 35.9% (95% CI 23.4–50.7%) for enteropathy-type T-cell lymphoma (ETTL) to 65.8% (95% CI 54.0–75.9%) for anaplastic large cell lymphoma (ALCL). The 5-year OS was 38.5% (95% CI 35.5–41.6%) for all PTCL patients and ranged from 20.3% (95% CI 12.5–31.2%) for ETTL to 56.5% (95% CI 42.8–69.2%) for ALCL. These data suggest that there is marked heterogeneity across PTCL subtypes in the benefits of anthracycline-based chemotherapy. While anthracyclines produce CR in half of PTCL patients, this yields reasonable 5-year OS for patients with ALCL but not for those with PTCL-NOS or ETTL. Novel agents and regimens are needed to improve outcomes for these patients.

## 1. Introduction

Peripheral T-cell lymphoma (PTCL) is a heterogeneous group of non-Hodgkin's lymphomas (NHL) characterized by poor treatment outcome with conventional chemotherapy. Anthracycline-based chemotherapy remains the standard treatment for patients with PTCL although such regimens have failed to induce sustained remissions for most patients. The role of anthracyclines in the treatment of PTCL remains debatable. The International PTCL Clinical and Pathologic Review Project retrospectively demonstrated no difference in overall survival (OS) comparing patients who did or did not receive an anthracycline for PTCL [1]. Prior studies have established worse outcome for PTCL compared to aggressive B-cell NHL treated with anthracycline-based chemotherapy, in terms of response, relapse, and OS rates [2–4]. Since there are no large randomized prospective studies that compare the benefits of anthracycline-based therapies to other strategies, we conducted a systematic literature review and meta-analysis of first-line therapy for PTCL patients to elucidate the role of anthracyclines and examine the complete response (CR) and OS rates associated with anthracycline-based regimens.

Given the well established favorable outcomes for patients with anaplastic lymphoma kinase (ALK) positive anaplastic large cell lymphomas (ALCL) [5], along with the heterogeneity in response and survival rates across PTCL subgroups, we focused our analyses on non-ALCL PTCL and performed subgroup meta-analyses on the outcomes of anthracycline-based regimens for patients with PTCL- not otherwise specified (NOS), angioimmunoblastic T-cell lymphoma (AITL), natural-killer/T-cell (NK/T-cell) NHL, and enteropathy-type T-cell lymphoma (ETTL) subtypes. 

## 2. Methods

### 2.1. Systematic Literature Review

Studies were identified by searching Medline and Google Scholar databases through 2010 and the conference proceedings of the American Society of Hematology and the American Society of Clinical Oncology for the years 2003 to 2010. Each search used combinations of the terms “Peripheral T-Cell Lymphoma,” “T-Cell Lymphoma,” “Anthracyclines,” “CHOP,” “Doxorubicin,” “Mitoxantrone,” “Daunorubicin,” “CVAD,” and “Adriamycin.” Two reviewers (A. N. AbouYabis and P. J. Shenoy) performed study selection, quality assessment, and data extraction independently using standardized forms. Any disagreement was resolved by a third reviewer (C. R. Flowers or M. J. Lechowicz).

### 2.2. Meta-Analysis Inclusion Criteria, Study Selection, and Data Extraction

Criteria for including studies in the meta-analysis were (1) studies involving patients with untreated PTCL (studies involving relapsed/refractory PTCL patients were included only if they provided separate outcome data for untreated PTCL patients), (2) treatment with anthracycline-based regimen, (3) reporting in English, and (4) reporting of CR rates and/or 5-year OS. Only full text reports were included as most abstracts presented preliminary results with a short followup. The primary outcome measures were OS and CR. Extracted data also included type of study (prospective/retrospective), PTCL subtype, pretreatment disease status, and median follow-up time. Studies were carefully screened for possible duplication of study population based on the participating institutions and period of presentation of patients. Additional studies not included in the meta-analysis were discussed in the narrative review.

### 2.3. Data Analysis and Statistical Methods

Studies included in the subtype-specific and combined PTCL meta-analyses were evaluated for heterogeneity as described below and evaluated for suitability for pooling. Pooled estimates of the CR rate and the 5-year OS for patients treated with anthracycline-containing regimens were computed. DerSimonian and Laird random effects [6] and Mantel-Haenszel fixed effect models [7] were used to combine subgroups to determine the overall effects. For each analysis, a forest plot was generated to display results. The study-to-study variance (tau-squared) was not assumed to be the same for all subgroups; this value was computed within subgroups and not pooled across subgroups. The consistency of results (effect sizes) among studies was investigated by means of two heterogeneity tests, the *χ* ^2^-based Cochran's *Q* test, and the *I* ^2^ statistic. To evaluate heterogeneity across reported results, we performed visual inspection of forest plots and compared the fixed effects and random effects models.

We considered that heterogeneity was present when the *P* value of the Cochran's *Q* test was <.1 and *I* ^2^ statistic was >50%. Potential publication bias was estimated with the Begg and Mazumdar rank correlation test [8], Egger's test of intercept [9], and Duval and Tweedie's trim and fill test [10]. We also evaluated whether our estimates of CR and 5-year OS were influenced by publication bias by assessing funnel plots of the logit of the estimate versus its standard error and by comparing pooled estimates of CR and 5-year OS rates for full text reports and studies reported as abstracts only. Tests for publication bias were repeated after inclusion and exclusion of abstracts published during that timeframe but not later published as full manuscripts to determine if adding abstracts in the meta-analyses changed the results and added significant value.

Univariate metaregression analyses were conducted to identify patient characteristics (age > 60 years, male gender, stage III/IV, B symptoms, extranodal disease, LDH >upper limit of normal, ECOG performance status ≥ 2, bone marrow involvement, and high/high intermediate International prognostic index score) that were significant predictors of CR rate and 5-year OS. A two-sided alpha error of 0.05 was used to declare statistical significance. Statistical analyses were performed using SAS software, Version 9.1.3 (SAS Institute Inc., Cary, NC, USA) and Comprehensive Meta Analysis, Version 2 (Biostat Inc.). 

## 3. Results

**Figure 1:**
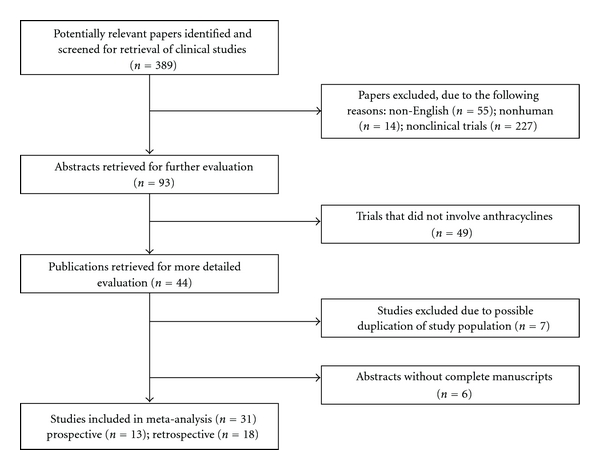
Quorum flow chart of study inclusion. Illustration of the number of articles identified in literature search and reasons for exclusion. Thirty studies met the inclusion and exclusion criteria.

Overall, 389 potentially relevant references describing initial treatments for PTCL were identified and screened for retrieval. Among these, 44 studies involved first-line therapy with anthracyclines; six studies were excluded due to possible duplication of study population, and six studies published as abstracts only were excluded.  Figure 1 depicts the exclusion of articles. Thirty-one studies (13 prospective and 18 retrospective) that involved 2,815 patients and met all the inclusion and exclusion criteria were included in the meta-analyses (Tables 1 and 2). 

### 3.1. Systematic Literature Review

#### 3.1.1. Response to Anthracycline Regimens across PTCL Subtypes

The CR rates associated with anthracycline-based regimens ranged from 30% to 76% across studies [2, 4, 12, 23, 24, 27–29, 32, 38, 39]. As expected, ALK-positive ALCL showed a higher CR rate with anthracycline-based chemotherapy than other T-cell lymphomas [4, 40]. Within the non-ALCL PTCL subgroups, treatment with cyclophosphamide-doxorubicin-vincristine-prednisone (CHOP) or CHOP-like chemotherapy produced CR in 36–70% of AITL patients, in 44–64% of PTCL-NOS patients, and in 33% of ETTL patients [24, 32, 36, 39]. 

NK/T-cell NHL demonstrated CR rates ranging between 40% and 70% in localized disease and a CR rate of 25% in advanced disease [22, 26, 33]. In patients with stage I/II disease, radiotherapy (RT) improved CR rates to 52–100% [25, 30, 31, 35, 37, 41–44]. Given the high rate of disease progression during standard CHOP chemotherapy (35%) as well as the previously documented superiority of CHOP-14 over standard CHOP by the German high-grade NHL study group [45, 46], two cycles of dose-intense CHOP every two weeks (DI-CHOP-14) followed by RT and consolidation therapy with four cycles of standard CHOP thereafter was investigated by Kim et al. and Lee et al. in patients with localized nasal NK/T-cell NHL [18, 47]. Overall, this regimen improved the CR rate to 76%, as compared to the 58% achieved by standard CHOP-21. The authors believed that this approach also reduced locoregional failure, likely due to early institution of RT [18, 47]. 

#### 3.1.2. CHOP versus More Intensive Anthracycline-Based Regimens

There have been conflicting data regarding the benefits of intensive anthracycline-based chemotherapy regimens compared with CHOP in the first-line treatment of PTCL and other aggressive NHLs. A randomized phase 3 trial demonstrated no significant difference in the partial response, CR rates, or 3-year OS comparing CHOP and three intensive chemotherapy regimens for all patients with aggressive NHL [48]. The prospective “LTP95” protocol showed no difference in event-free survival (EFS) or OS between CHOP and vinblastine-ifosfamide-cisplatin and doxorubicin-bleomycin-vinblastine-dacarbazine regimen [49]. In a retrospective study comparing cohorts of PTCL patients, excluding ALCL, the CR rates were similar between CHOP and more intensive regimens, including hyper-CVAD (58% versus 59%, *P* = .99), with an estimated 3-year OS rates of 43% and 49%, respectively [34]. There has, however, been one randomized trial showing an 18% difference in disease-free survival (DFS) at 5 years (*P* = .0002) favoring intensive chemotherapy over CHOP [50].

#### 3.1.3. Relapse following Anthracycline Regimens

PTCL patients treated with anthracycline-based regimens generally relapse at a higher rate than patients with B-NHL (43% versus 29%, *P* < .001) and have a significantly shorter freedom-from-relapse survival (median: 34 months versus not reached for B-cell; *P* = .002) [4]. Moreover, disease progression during chemotherapy occurred in 30%–40% of patients [26, 32, 47, 51]. Relapse rates for PTCL varied by subtype [24, 36]. A study by the British Columbia Cancer Agency showed more favorable 5-year progression-free survival (PFS) for ALCL and PTCL-NOS subtypes (28%) followed by ETTL (22%), NK/T-cell, and AITL subtypes (13–15%) [32]. 

#### 3.1.4. Differences in Survival across PTCL Subtypes

Five-year OS ranged between 63% and 75% for ALCL versus 26%–36% for non-ALCL subtypes [4, 24, 29, 40, 52]. Among non-ALCL PTCL subtypes, the 5-year OS ranged between 28% and 36% in AITL, 45% in PTCL-NOS, and <25% in patients with ETTL [24, 32, 36]. For NK/T-cell NHL, 5-year OS was 25%–77% depending on the treatment modality [26, 31–33, 35, 43]. Chemotherapy plus RT resulted in the same 5-year OS as RT, and both modalities were superior to chemotherapy alone [26, 31, 33, 35, 37, 43].

### 3.2. Meta-Analyses

#### 3.2.1. Complete Remission Rates

In order to determine if pooling the studies for subtype-specific meta-analysis and as an entire PTCL group was appropriate, we first determined the *P* value for the Cochran's *Q* test and the *I* ^2^ statistic for the studies to identify and quantify the level of heterogeneity [53, 54].

When combining studies to estimate CR, the *P* value for the Cochran's *Q* test was <.01 and the *I* ^2^ statistic was 59% indicating substantial heterogeneity [53]; hence, a pooled estimate of the CR rate for all PTCL patients was not calculated. In the subtype-specific analysis, AITL, ALCL, and NK/T nasal demonstrated no evidence of heterogeneity (all *I* ^2^ < 50%) and hence were pooled for subtype-specific meta-analysis. 

**Figure 2:**
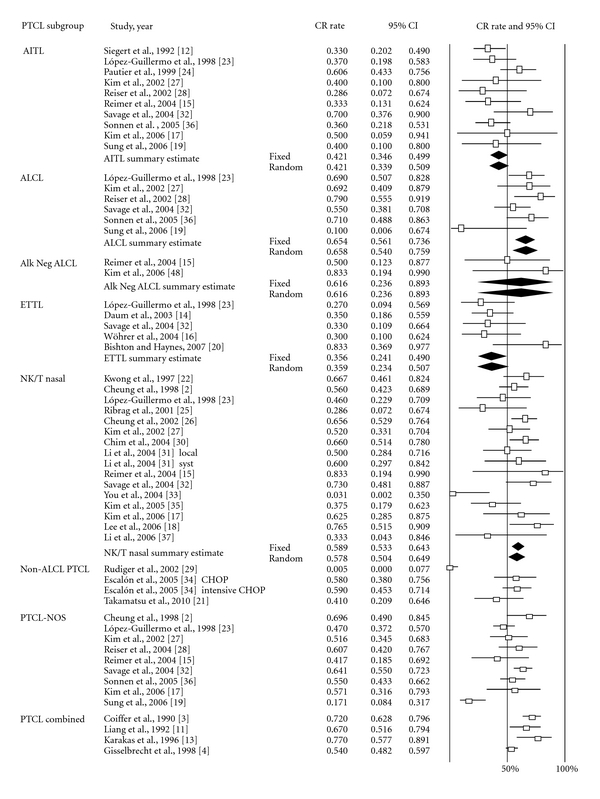
Meta-analysis of complete response rate of patients undergoing anthracycline-based chemotherapy by PTCL subtype. Forest plot of the complete response rate along with summary estimates and its 95% CI in diamonds. Horizontal lines show the 95% CI for each trial. Only subtypes showing no evidence of heterogeneity were grouped. Within each subtypes, studies were ordered by year of reporting and alphabetical order. Squares on the plot are proportional to the weight of each study. Fixed effect and random effects models summary estimates are depicted in boldface.

Analyses by PTCL subtype (Figure 2, black diamonds) revealed the following CR rates: ALCL 65.8% (95% CI 54.0–75.9%), NK/T 57.8% (95% CI 50.4–64.9%), AITL 42.1% (95% CI 33.9–50.9%), and ETTL 35.9% (95% CI 23.4–50.7%). For NK/T cell lymphoma, the CR rates for treatment with chemotherapy alone and combination chemoradiation therapy were 57.1% (95% CI 48.8–64.9%) and 68.3% (95% CI 60.7–75.0%), respectively. To address the disparate outcomes in ALK-positive ALCL, we performed a meta-analysis of studies removing data for this PTCL subset. With ALK-positive ALCL patients excluded, the estimated CR rate for PTCL patients receiving anthracycline-based chemotherapy (*n* = 1,191) was 50.1% (95% CI 44.9–55.3%).

**Figure 3:**
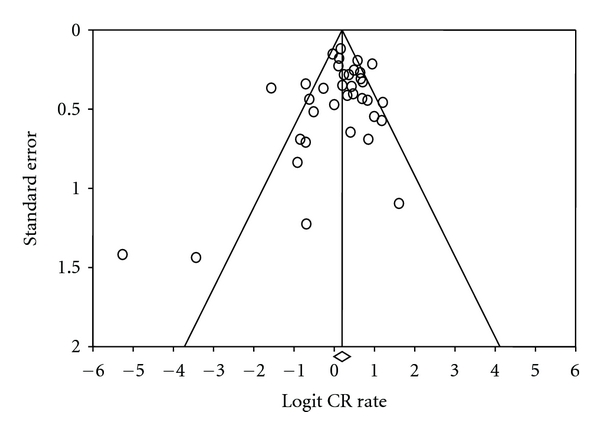
Funnel plot of standard error by logit complete response rate. The funnel plot of studies included in the meta-analysis of complete response rate illustrates the standard error and logit of complete response rate.

The funnel plot (Figure 3) of studies included in the meta-analysis of CR rates appeared symmetrical. Begg and Mazumdar's rank correlation test (*P* = .16), Egger's test of intercept (*P* = .16), and Duval and Tweedie's trim and fill test (no studies added) all indicated that there was no clear evidence of publication bias. Pooled CR estimates of abstracts and full text reports were not significantly different (*P* = .49). Tests for publication bias repeated after inclusion of abstracts showed that adding abstracts did not add any significant informational value. The univariate metaregression analysis of prospective studies revealed that age at diagnosis > 60 (*P* = .03), the presence of stage III/IV (*P* = .046), B-symptoms (*P* = .012), and high/high-intermediate IPI (*P* = .056) were significant predictors of lower CR rates (Table 3).

#### 3.2.2. Five-Year Survival Rates

**Figure 4:**
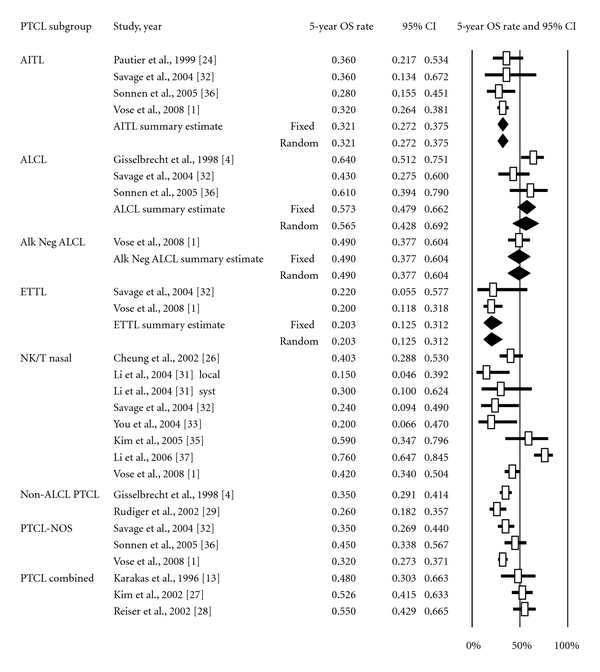
Meta-analysis of 5-year overall survival rate of patients undergoing anthracycline-based chemotherapy by PTCL subtype. Forest plot of the complete response rate along with summary estimates and its 95% CI in diamonds. Horizontal lines show the 95% CI for each trial. Only subtypes showing no evidence of heterogeneity were grouped. Within each subtypes, studies were ordered by year of reporting and alphabetical order. Squares on the plot are proportional to the weight of each study. Fixed effect and random effects models summary estimates are depicted in boldface.

Of the studies included in the meta-analyses for 5-year OS, only two were prospective studies [4, 13]. The *P* value for the Cochran's *Q* test was <.01 and the *I* ^2^ statistic was 78% indicating substantial heterogeneity [53]; hence, a pooled estimate of 5-year OS for all PTCL patients was not calculated. Among the subtypes, studies reporting results for AITL, ALCL, and ETTL demonstrated no evidence of heterogeneity (all *I* ^2^ = 0%) and hence were pooled for subtype-specific meta-analysis. The pooled 5-year OS estimates for PTCL subtypes demonstrating no evidence of heterogeneity were as follows: ALCL 56.5% (95% CI 42.8–69.2%), AITL 32.1% (95% CI 27.2–37.5%), and ETTL 20.3% (95% CI 12.5–31.2%) (Figure 4). When ALCL was excluded, the 5-year OS for all PTCL patients (*n* = 1,691) was 36.6% (95% CI 31.5–42.0%).

**Figure 5:**
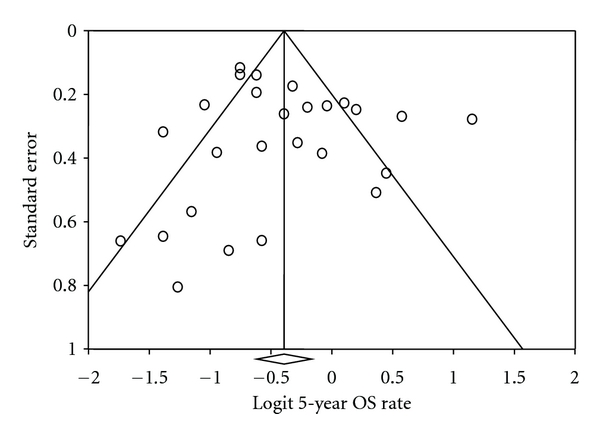
Funnel plot of standard error by logit 5-year overall survival rate. The funnel plot of studies included in the meta-analysis of 5-year overall survival rate illustrates the standard error and logit of 5-year overall survival rate.

The funnel plot (Figure 5) of studies included in the meta-analysis of 5-year OS rates showed wide variance but was predominantly symmetrical. The Begg and Mazumdar's rank correlation test (*P* = .50), Egger's test of intercept (*P* = .26), and Duval and Tweedie's trim and fill test (no studies added) all indicated that there was no clear evidence of publication bias. Pooled 5-year OS estimates of abstracts and full text reports were not significantly different (*P* = .87). Tests for publication bias repeated after inclusion of abstracts showed that adding abstracts did not add any significant value. 

## 4. Discussion

Despite the established worse outcome of anthracycline-based chemotherapy in PTCL patients compared to aggressive B-cell NHL [2–4], such regimens, especially CHOP, have remained the standard treatment for PTCL. In this meta-analysis, the CR rate achieved with anthracycline-based regimens ranged from 36% in ETTL to 66% in ALCL. Five-year OS across PTCL subtypes also ranged widely from 20% in ETTL to 57% in ALCL. As has already been established [4, 40], ALCL patients had a markedly better 5-year OS than other PTCL patients (57% versus 37%, *P* < .001). However, it is worth noting here that the majority of studies did not report on the ALK status of ALCL patients. Results on ALK-negative and ALK-positive ALCL patients were grouped together in most studies. Only three studies that reported on ALK-negative ALCL had sufficient data to be included in the meta-analysis [1, 15, 17] and revealed a CR rate of 62% and a 5-year OS of 49% [1] in this subgroup. Although ALK-negative ALCL has poorer outcomes when compared to ALK-positive ALCL, both subsets of ALCL appear to have superior outcomes when compared to other PTCL subtypes.

### 4.1. Study Limitations

Until recently, the majority of our knowledge about treatment outcomes in PTCL emerged from small phase 2 studies. Hence, the data included in this meta-analysis comes from phase 2 clinical trials with all the inherent drawbacks of such studies. Additionally, the low prevalence of PTCL and its heterogeneity makes it difficult to draw conclusions from most of these small individual studies. It should also be noted that there were no consistent standards for reporting results. This limited the number of studies eligible for inclusion. Moreover, most studies did not specify the timing and criteria for CR determination and utilized investigator determination of CR rather than central review. This might lead to overestimate of the CR rate. Some studies did not report CR and OS for PTCL subtypes separately. These contributed to the review of overall CR and 5-year OS rates but not to subtype estimates. 

High-quality meta-analyses often utilize data from randomized, double-blinded, controlled trials with descriptions of dropouts and withdrawals [55]. We did not perform Jadad scoring of individual trials because PTCL and its subtypes are rare diseases that precluded performance of large randomized trials. While, in an ideal setting, a meta-analysis for PTCLs would utilize data from randomized phase 3 trials, our meta-analyses represent a review of the best available data from existing publications on the treatment of PTCL.

Another concern raised by previous studies is the heterogeneity of PTCL populations in the published literature. This was addressed by separating out PTCL subtypes before computing estimates of CR and 5-year OS rates. Moreover, there was heterogeneity in reporting of data across studies. The meta-analysis of CR rate for PTCL, NOS, and PTCL as a whole group was associated with moderate heterogeneity (*I* ^2^ 71% and 59%, resp.) as was the meta-analysis of 5-year OS rate for PTCL and NOS (*I* ^2^ = 54%), while for NK/T-cell, nasal lymphoma, and PTCL as a whole group was associated with high degree of heterogeneity (*I* ^2^ > 75%). To address this, random effects models were used but this degree of inconsistency may yield instability in the results. Nevertheless, these analyses represent the best available data for these rare disease entities and may only be improved by larger randomized controlled trials which have not been performed to date and may not be practical for some PTCL subsets.

We attempted to estimate the PFS rates achieved with anthracycline-based regimens, but while some authors reported PFS rates, others presented DFS or EFS rates without consistent definitions of events precluding combination. Unfortunately, the international prognostic index was not reported for the majority of patients participating in the included trials. However, whenever available, we extracted patient data and included it in the metaregression of predictors of CR shown in Table 3. Moreover, some studies included in the meta-analysis of CR rates were presentations of early results and had insufficient followup to be included in the meta-analysis of 5-year OS.

As in any meta-analysis, there was concern for publication bias. We addressed this issue using funnel plots and statistical testing and did not find evidence suggestive of overt publication bias. We also compared analysis with the addition of meeting abstracts that were not published as full manuscripts and determined that adding these studies did not influence our results. 

Since retrospective studies are subject to selection, recall, and other biases, we also analyzed prospective studies separately. When only prospective studies included in the meta-analysis of CR were examined, PTCL as a whole group demonstrated substantial heterogeneity (*I* ^2^ = 69%), AITL, ALK-negative ALCL, and NK/T demonstrated no evidence of heterogeneity (all *I* ^2^ = 0%), and ETTL demonstrated moderate heterogeneity (*I* ^2^ = 50%). The estimated CR rate for PTCL subtypes when analyses are limited to prospective studies were as follows: NK/T 72.7% (95% CI 53.3–86.1%), ALK-negative ALCL 61.6% (95% CI 23.6–89.3%), ETTL 43.3% (95% CI 20.1–69.8%), and AITL 34.3% (95% CI 23.3–47.4%), which differ somewhat from the response rates reported above. Since there were only two prospective studies reporting 5-year OS, separate analysis was not performed.

### 4.2. Summary of Findings

#### 4.2.1. Is There a Benefit for Anthracyclines in the Treatment of PTCL Patients outside of the ALCL Subsets?

To answer this question it may be useful to look at the results of anthracycline-based regimens in the treatment of other NHL subtypes where their benefit has already been established. While the CR rate of 52% from this meta-analysis achieved with anthracyclines in the treatment of PTCL patients (excluding ALK-positive ALCL) seems comparable to the 44%–63% CR rates obtained with CHOP chemotherapy in diffuse large B-cell lymphoma (DLBCL) patients in the pre-rituximab era [48, 56], the estimated 5-year OS for PTCL patients was only 35% compared to the 41% in intermediate-grade NHL in general [57] and 45%–70% in DLBCL [58–60]. Moreover, the International PTCL Clinical and Pathologic Review Project retrospectively demonstrated no difference in OS comparing patients who did and did not receive an anthracycline for any subtype of PTCL [1].

#### 4.2.2. What Modifications to the Current Anthracycline-Based Regimens Could Improve Treatment Outcomes in PTCL Patients?

Modifications in dose density and intensity of CHOP-type regimens (like DI-CHOP) may overcome the high rate of disease progression and induce better long-term responses in PTCL [18, 47]. The German high-grade NHL study group has previously shown that reducing the interval between CHOP cycles from 21 days to 14 days improves EFS [45] as well as OS [45, 46]. Another recent approach to improve efficacy of CHOP regimen was substituting doxorubicin with pegylated liposomal doxorubicin, which was suggested to persist in the blood circulation significantly longer than doxorubicin [61].

**Addition of Other Active Agents?**

Other modifications to the CHOP-based chemotherapy include the addition of etoposide [45, 46], bortezomib, and purine analogs [17, 62–68]. Although the addition of etoposide to CHOP-based regimens improved CR in T-NHL patients in some studies, EFS and OS rates have been disappointing when compared to B-NHL [13, 69, 70]. On the other hand, encouraging results have been achieved adding gemcitabine to CHOP-etoposide in the front-line setting [17, 68]. Another approach to overcome drug resistance in PTCL is the combination of CHOP with the proteasome inhibitor bortezomib. This currently is being investigated in a phase 1/2 study in patients with advanced stage PTCL [71].

Another option for improving the outcome of anthracycline-based chemotherapy in PTCL is combining the anti-angiogenic agent bevacizumab with CHOP (A-CHOP). The basis of this combination emerges from the finding that serum concentrations of the vascular endothelial growth factor (VEGF) have an independent prognostic influence on survival in NHL [72]. Moreover, significant expression of VEGF transcripts was observed in PTCL, particularly in AITL [73]. This combination has been studied in the Eastern Cooperative Oncology Group (ECOG).

**Incorporation of Immunotherapy?**

The significant improvements in response rates and survival achieved by adding rituximab to CHOP-type chemotherapy in DLBCL [56, 60, 74] triggered the investigation of similar chemo-immunotherapy combinations in PTCL. The interim analysis of the CONCEPT phase 2 study of denileukin diftitox in combination with CHOP revealed a 68% overall response rate and 57% CR in patients with PTCL [75]. Long-term results are still awaited. Alemtuzumab-chemotherapy combinations also have been evaluated in several studies [76–78]. Alemtuzumab-CHOP combinations in the front-line treatment of non-ALCL PTCL patients resulted in 60–80% CR rate with estimated 2-year OS of 53–75% [79]. The estimated 2-year EFS in those studies ranged between 40% and 50%. Moreover, the combination of alemtuzumab with fludarabine-cyclophosphamide-doxorubicin induced remission in 63% (58% CR) of ALK-negative PTCL patients treated in the front-line setting [80]. However, infections have been a concern in all alemtuzumab-combination studies.

**Incorporation of Radiation Therapy?**

The addition of RT also may be important particularly in NK/T cell lymphoma. Our meta-analysis revealed that anthracycline-based chemotherapy alone resulted in a CR rate of 49% (95% CI 38.7–59.5%) while chemoradiotherapy resulted in CR of 59.6% (95% CI 42–75%; *P* = .02). However, these results cannot be used as strong evidence to the benefits of combination of chemoradiotherapy in NK/T-cell lymphoma over chemotherapy alone, since most studies included in this meta-analysis did not report treatment results in relation to the extent and stage of the disease [18, 26, 30, 31, 33, 35, 37, 43, 47, 81]. Clarifying the benefits of chemoradiotherapy over chemotherapy in NK/T cell NHL will require larger randomized controlled clinical trials.

**Consolidating Responses with Hematopoietic Stem Cell Transplant?**

Since the major difficulty in the treatment of PTCL with conventional chemotherapy has been sustaining CR [3, 23, 27, 29, 39], autologous and allogeneic hematopoietic stem cell transplant have been explored as ways to consolidate remissions and prolong survival among PTCL patients treated with anthracycline-based regimens. However, there has been conflicting data regarding the advantage of performing transplantation in first remission in PTCL [82–90].

#### 4.2.3. What Are the Alternatives to Anthracyclines in PTCL Management?

In the search for nonanthracycline alternatives, some agents have demonstrated promise as single agents in NHL in general and PTCL in particular. Of those are the anti-folate agents, such as pralatrexate, which showed 28% overall response rate in patients with relapsed/refractory PTCL and 49% rate of disease control leading to its FDA approval in this setting [91]. Moreover, the activity of purine nucleoside analogues such as pentostatin, nelarabine, and gemcitabine appears promising as well [62–67, 92]. Histone deacetylase (HDAC) inhibitors, such as vorinostat and romidepsin, are another promising group of agents that are being tested as single agents or in combination in treatment of PTCL both front-line and relapsed/refractory settings [93, 94]. Although nonanthracycline-based chemotherapy seems an attractive alternative, comparative studies are needed to establish its benefit over anthracyclines in PTCL. 

## 5. Conclusion

Despite achieving CR with front-line anthracycline-based therapy in more than half of PTCL patients, 5-year OS remains poor for most PTCL subtypes. Although CHOP remains the standard front-line therapy for PTCL, given these poor outcomes, most PTCL patients (with the exception of ALK-positive ALCL) should be considered for clinical trials of initial therapy. Future clinical trials need to focus on subtype-specific treatment, incorporation of newer agents, and nonanthracycline-based combinations to improve the long-term outcome for PTCL patients. Moreover, strategies capable of sustaining responses such as maintenance therapy and transplant consolidation should be actively investigated in prospective clinical trials. Given the small number of PTCL patients seen by any single institution, such trials need to be multicentered and community oncologists should encourage their patients to enroll in such studies whenever available. 

##  Disclosure

Dr. M. J. Lechowicz has been a consultant for the Allos Therapeutics and Eisai in the past. Dr. M. J. Lechowicz has also participated in clinical trials using pralatrexate, romidepsin, and denileukin diftitox. The authors alone are responsible for the content and writing of the paper.

## Figures and Tables

**Table 1:** Prospective studies included in meta-analyses.

Study, Year	Regimen	PTCL subgroup*	*n*	CR%	OS
Coiffer et al., 1990 [3]	ACVB (LNH84)	PTCL-combined	108	72.0	
Liang et al., 1992 [11]	BACOP ± Methotrexate	PTCL-combined	42	67.0	3 yr 52%
Siegert et al., 1992 [12]	Pred ± COPBLAM/IMVP	AITL	39	33.0	3 yr 41%
Karakas et al., 1996 [13]	VACPE	PTCL-combined	27	77.0	5 yr 48%
Gisselbrecht et al., 1998 [4]	Anthracycline-based	PTCL-combined	288	54.0	5 yr 41%
		ALCL	60	72.0	5 yr 64%
		Non-ALCL PTCL	228	49.0	5 yr 35%
Daum et al., 2003 [14]	CHOP	ETTL	23	35.0	2 yr 49%
Reimer et al., 2004 [15]	CHOP	PTCL-combined	30	43.3	
		PTCL-NOS	12	41.7	
		AITL	12	33.3	
		ALK Neg ALCL	4	50.0	
		NK/T nasal	2	100.0	
Wöhrer et al., 2004 [16]	CHOEP	ETTL	10	30.0	
Kim et al., 2006 [17]	CHOP-EG	PTCL-combined	26	61.5	1 yr 70%
		PTCL-NOS	14	57.1	
		AITL	2	50.0	
		ALK Neg ALCL	2	100.0	
		NK/T nasal	8	62.5	
Lee et al., 2006 [18]	DI-CHOP + RT	NK/T nasal	17	76.5	3 yr 67%
Sung et al., 2006 [19]	CEOP-B	PTCL-combined	52	17.3	
		PTCL-NOS	41	17.1	
		AITL	5	40.0	
		ALCL	4	0.0	
Bishton and Haynes, 2007 [20]	HDCT + PBSCT	ETTL	6	83.3	
Takamatsu et al., 2010 [21]	THP-COP	Non-ALCL PTCL	17	41.0	3 yr 35%

PTCL: peripheral T-cell lymphoma; n: number of patients; CR: complete response; OS: overall survival; ACVB: doxorubicin, cyclophosphamide, vindesine, bleomycin, and prednisone; BACOP: Bleomycin, adriamycin, cyclophosphamide, vincristine, and prednisone; Pred, prednisolone; COPBLAM: cyclophosphamide, vincristine, and prednisone with bleomycin, doxorubicin, and procarbazine; IMVP: ifosfamide, methotrexate, and etoposide; VACPE: vincristine, doxorubicin, cyclophosphamide, prednisone, and etoposide; NK/T nasal, NK/T-cell lymphoma nasal type; NOS: not otherwise specified; ALCL: anaplastic large cell lymphoma; AITL: angioimmunoblastic T-cell lymphoma; ETTL: enteropathy-type T-cell lymphoma; CHOP: cyclophosphamide, doxorubicin, vincristine, and prednisone; RT: radiation therapy; CMT: combined modality treatment; V: vincristine; syst, systemic; Neg: negative; CHOEP: cyclophosphamide, doxorubicin, etoposide, vincristine, and prednisone; CT: chemotherapy; CVAD: cyclophosphamide, doxorubicin, vincristine, and dexamethasone; VIP: vinblastine, ifosfamide, and cisplatin; ABVD: doxorubicin, bleomycin, vinblastine, and dacarbazine; EG: etoposide and gemcitabine; DI: dose-intensive; CEOP-B: cyclophosphamide, epirubicin, vincristine, prednisone, and bleomycin; HDCT: high-dose chemotherapy; PBSCT: peripheral blood stem cell transplant; THP-COP: pirarubicin, cyclophosphamide, vincristine, and prednisone.

*PTCL-combined represents the whole group of PTCL patients. In cases where studies separated ALK-positive and ALK-negative ALCL and provided separate results for the two subgroups, ALK-positive patients were excluded from the meta-analysis. Non-ALCL PTCL indicates PTCL without any ALCL subtype included.

**Table 2:** Retrospective studies included in meta-analyses.

Study, Year	Regimen	PTCL subgroup*	*n*	CR%	OS
Kwong et al., 1997 [22]	Anthracycline-based	NK/T nasal	24	66.7	
Cheung et al., 1998 [2]	Anthracycline-based	PTCL-NOS	24	69.6	2 yr 63%
		NK/T nasal	51	56.0	2 yr 43%
Löpez-Guillermo et al., 1998 [23]	Anthracycline-based	PTCL-combined	174	49.0	4 yr 38%
		PTCL-NOS	95	47.0	4 yr 32%
		ALCL	30	69.0	
		AITL	22	37.0	
		NK/T nasal	14	46.0	
		ETTL	12	27.0	
Pautier et al, 1999 [24]	CHOP-like	AITL	33	60.6	5 yr 36%
Ribrag et al., 2001 [25]	CHOP-type ± RT	NK/T nasal	7	28.6	
Cheung et al., 2002 [26]	CMT	NK/T nasal	61	65.6	5 yr 41%
Kim et al., 2002 [27]	CHOP ± RT	PTCL-combined	78	52.6	5 yr 36%
		ALCL	13	69.2	
		PTCL-NOS	31	51.6	
		AITL	5	40.0	
		NK/T nasal	25	52.0	
Reiser et al., 2002 [28]	Anthracycline-based	PTCL-combined	66	62.0	5 yr 55%
		ALCL	19	79.0	
		PTCL-NOS	28	60.7	
		AITL	7	28.6	
Rüdiger et al., 2002 [29]	Adriamycin-based	Non-ALCL PTCL	96		5 yr 26%
Chim et al., 2004 [30]	Anthracycline-based + RT	NK/T nasal	47	65.9	
Li et al., 2004 [31]	CHOP-based	NK/T nasal local	18	50.0	5 yr 15%
	CHOP-based + RT	NK/T nasal local	27	74.1	5 yr 59%
	CHOP-based	NK/T nasal syst	10	60.0	5 yr 30%
	CHOP-based + RT	NK/T nasal syst	10	30.0	5 yr 20%
Savage et al., 2004 [32]	CHOP-type	PTCL-NOS	117	64.1	5 yr 35%
		ALCL	33	55.0	5 yr 43%
		AITL	10	70.0	5 yr 36%
		NK/T nasal	17	73.0	5 yr 24%
		ETTL	9	33.0	5 yr 22%
You et al., 2004 [33]	CT + RT	NK/T nasal	16		5 yr 42%
	CT	NK/T nasal	15		5 yr 20%
Escalón et al., 2005 [34]	CHOP	Non-ALCL PTCL	24	58.0	3 yr 43%
	CHOP Intensive	Non-ALCL PTCL	52	59.0	3 yr 49%
Kim et al., 2005 [35]	CHOP/COPBLAM-V + RT	NK/T nasal	16	37.5	5 yr 59%
	RT	NK/T nasal	33	52.0	5 yr 76%
Sonnen et al., 2005 [36]	CHOP-type	PTCL-combined	125	53.0	5 yr 43%
		ALCL	21	71.0	5 yr 61%
		PTCL-NOS	70	55.0	5 yr 45%
		AITL	34	36.0	5 yr 28%
Li et al., 2006 [37]	CHOP-based + RT	NK/T nasal	71	84.5	5 yr 76%
	CHOP-type alone	NK/T nasal	3	33.3	
Vose et al., 2008 [1]	Anthracycline-based	PTCL-NOS	340		5 yr 32%
		ALK Neg ALCL	72		5 yr 49%
		AITL	243		5 yr 32%
		NK/T nasal	136		5 yr 42%
		ETTL	62		3 yr 20%

PTCL: peripheral T-cell lymphoma;* n*: number of patients; CR: complete response; OS: overall survival; ACVB: doxorubicin, cyclophosphamide, vindesine, bleomycin, and prednisone; BACOP: Bleomycin, adriamycin, cyclophosphamide, vincristine, and prednisone; Pred, prednisolone; COPBLAM: cyclophosphamide, vincristine, and prednisone with bleomycin, doxorubicin, and procarbazine; IMVP: ifosfamide, methotrexate, and etoposide; VACPE: vincristine, doxorubicin, cyclophosphamide, prednisone, and etoposide; NK/T nasal, NK/T-cell lymphoma nasal type; NOS: not otherwise specified; ALCL: anaplastic large cell lymphoma; AITL: angioimmunoblastic T-cell lymphoma; ETTL: enteropathy-type T-cell lymphoma; CHOP: cyclophosphamide, doxorubicin, vincristine, and prednisone; RT: radiation therapy; CMT: combined modality treatment; V: vincristine; syst, systemic; Neg: negative; CHOEP: cyclophosphamide, doxorubicin, etoposide, vincristine, and prednisone; CT: chemotherapy; CVAD: cyclophosphamide, doxorubicin, vincristine, and dexamethasone; VIP: vinblastine, ifosfamide, and cisplatin; ABVD: doxorubicin, bleomycin, vinblastine, and dacarbazine; EG: etoposide and gemcitabine; DI: dose-intensive; CEOP-B: cyclophosphamide, epirubicin, vincristine, prednisone, and bleomycin; HDCT: high-dose chemotherapy; PBSCT: Peripheral blood stem cell transplant.

*PTCL-combined represents the whole group of PTCL patients. In cases where studies separated ALK-positive and ALK-negative ALCL and provided separate results for the two subgroups, ALK-positive patients were excluded from the meta-analysis. Non-ALCL PTCL indicates PTCL without any ALCL subtype included.

**Table 3:** Metaregression of predictors of complete response rate.

Predictor variable	CR	
	Coefficient	*P* value
% age > 60	−0.029	.03
Gender, male	0.026	.27
% stage III/IV	−0.015	.046
% B symptoms	−0.036	.012
% extra-nodal disease	0.011	.25
% high LDH > ULN	−0.037	.26
% ECOG ≥ 2	−0.008	.46
% bone Marrow Positive	−0.026	.28
% high/high Inter IPI	−0.016	.056

Note: only prospective studies were included in the metaregression.
